# Estimation of potential agricultural non-point source pollution for Baiyangdian Basin, China, under different environment protection policies

**DOI:** 10.1371/journal.pone.0239006

**Published:** 2020-09-22

**Authors:** Yuan Tao, Jing Liu, Xiaoyan Guan, Haorui Chen, Xiaoqiang Ren, Shaoli Wang, Mengzhe Ji

**Affiliations:** 1 State Key Laboratory of Simulation and Regulation of Water Cycle in River Basin, China Institute of Water Resources and Hydropower Research, Beijing, China; 2 Department of Irrigation and Drainage, China Institute of Water Resources and Hydropower Research, Beijing, China; Institute for Advanced Sustainability Studies, GERMANY

## Abstract

To prevent and control non-point source pollution, many policies have been carried out by government in China. However, the effectiveness of these policies has rarely been evaluated. In this study, the potential and spatial distribution of agricultural non-point source pollution in the Baiyangdian Basin are reported. This investigation considers multiple parameters under various policies with county as a basic unit. The results for the potential pollution from chemical oxygen demand (COD), ammonia nitrogen (NH_3_-N), total nitrogen (TN) and total phosphorus (TP) are 60.89×10^4^, 3.93×10^4^, 87.05×10^4^ and 15.10×10^4^ Mg, with corresponding intensities of 190, 12, 272 and 47 kg ha^-1^ for the Baiyangdian Basin in 2016. The highest pollution from COD is attributed to livestock and poultry breeding, whereas TN and TP are dominantly produced by rural domestic sources, and NH_3_-N is mostly derived from planting. Spatially, distribution of the counties producing larger non-point source pollution presented a northeast to southwest direction, consistent with the Taihang mountain alignment in the basin. The counties with high pollution intensities are mostly in the south and east of the basin. Agricultural non-point source pollution control and prevention policies contributed in pollution reduction. Compared with 2016, the total potential pollution of COD, NH_3_-N, TN and TP in 2020 decrease by 45.1%, 14.7%, 37.9% and 37.4%, respectively, whereas for an assumed future time (F2), the decreases are 59%, 51.4%, 56.2% and 55.7%, respectively. Prevention measures should focus on reducing pollution from livestock and poultry breeding as well as planting.

## 1. Introduction

Agricultural non-point source pollution significantly contributes to water environment pollution in China [1]. These sources mainly include: fertilizer and pesticides, crops straws, livestock and poultry breeding, rural household solid wastes, rural domestic wastewater and rural human excrement and urine [2, 3]. Non-point source pollution of the environment poses an water environmental risk, irrespective of whether the pollutants can reach the waterbody by surface runoff or through soil leaching [4–6]. Pollution of a waterbody by surface runoff can cause eutrophication [7, 8], whereas leaching promotes accumulation of soil pollutants. The accumulated pollutants are partially transported into surface waterbodies by seepage, while other part portion infiltrating the soil cause groundwater contamination by ultimately leaching into the aquifer [9, 10]. To prevent agricultural non-point source pollution, proposed technologies fall into four including source reduction, blocking and interception in the process of transmission, nutrient recycling and ecological restoration of water body [11, 12]. In China, the Ministry of Agriculture and Rural Affairs (previously Ministry of Agriculture) emphasized the importance of agricultural non-point source pollution prevention and clearly stated the prevention tasks in 2015. Measures introduced for prevention included developing water-saving agricultural practices, pursuing a fertilizer and pesticide application zero-growth action strategy, ensuring comprehensive utilization of livestock and poultry breeding, and utilizing straw resources [13]. The application of these environmental protection policies has overall improved the environment [14].

The methods for estimating agricultural non-point source pollution are mainly classified as statistical empirical model and mechanical process model [3]. The most commonly used statistical empirical model is the export coefficient method, which involves the assessment of exporting different pollutants from the relationships of water quality parameters in typical regions. For large-scale studies, the export coefficients are usually of low accuracy or are hardly obtained [15]. Conversely, for the mechanical process model such as the Soil and Water Assessment Tool (SWAT), Agricultural Non-point Pollution Loading model (AGNPS) and Diffuse pollution estimation with remote sensing(DPeRS), high basic data are needed, and the parameters are difficult to calibrate and validate [16]. Therefore, distinguishing which method is better and which results are more reliable remains challenging, considering previous studies on the Baiyangdian Basin as examples.

In recent years, the agricultural non-point source pollution of Baiyangdian basin, its sub-catchment and Haihe basin which covers Baiyangdian basin has been estimated in many studies by different methods. In [5], the risk of nitrate leaching and nitrate runoff loss from intensive farmland in the Baiyangdian Basin (area of 3.2×10^6^ ha) was assessed using the distributed hydrological model SWAT. The results showed that the maximum nitrate nitrogen runoff loss from the farmland was 9.27 kg ha^-1^ in 2009 and 15.38 kg ha^-1^ in 1994 (with larger rainfall) happened in southern regions of the basin. Cui [17] adopted the statistical experience method for estimating the agricultural non-point source pollution load into rivers of the Baiyangdian Basin, reporting total nitrogen(TN), total phosphorus(TP), chemical oxygen demand(COD) and ammonia nitrogen (NH_3_-N) pollution were 6.22×10^4^, 1.85×10^4^, 0.76×10^4^ and 2.91×10^4^ Mg yr^-1^, respectively. In the study, chemical fertilizers and pesticides were reported as the main pollution sources. While Zhao [18] demonstrated that the non-point source pollution of TN loss into river in the Baiyangdian Basin was averaged just 793.2 Mg yr^-1^ during 2005–2010 based on the SWAT simulation. Moreover, Liu [19] calculated the agricultural non-point source pollution of a subregion of the Baiyangdian Basin (area of 3.9×10^5^ ha) by the SWAT, reporting TN and TP pollution of 20.51×10^4^ and 1.88×10^4^ Mg in 2011 and 31.11×10^4^ and 6.28×10^4^ Mg in 2013, respectively. Further, Qiu et al. [20] employed the pollution emission coefficient method to estimate the discharge of COD, TN, TP, and NH_3_-N discharges into the Haihe River Basin (area of 3.18×10^7^ ha) from rural domestic wastewater, livestock, and fertilizer in 2008. The total emission of COD, TN, TP, and NH_3_-N pollution were 243×10^4^, 304×10^4^, 54×10^4^, and 180×10^4^ Mg, respectively, with corresponding intensities of 111, 140, 30 and 83 Mg ha^-1^. Additionally, Wang [21] simulated the nitrogen and phosphorus loads in the Haihe Basin using SWAT and obtained loads of 5.26×10^4^ and 1.05×10^4^ Mg, respectively in 2015. Feng et al. [22] adopted DPeRS model in analyzing the agricultural non-point source pollution loss in the Haihe Basin and reported the emissions of TN and TP pollution of 13.62×10^4^ and 8152 Mg g, respectively, with corresponding amounts of 2.53×10^4^ and 1597 Mg discharged into river in 2016.

In above literatures, the minimal research element of existing studies using software were always sub-catchment which was a multi-county region. In China, agricultural non-point source pollution prevention was primarily government’s responsibility, with Ministry of Environmental Protection and the Ministry of Agriculture and Rural Affairs providing compulsory standards as well as the average targets for all over the country. Local governments were than required to develop detailed goals based on the requirements from the ministries, considering the agricultural non-point source pollution status of each administrative area. Generally, a county or district is the unit for actionable goals formulation, and hence, regarding a sub-catchment as a unit poses problems for implementing the pollution prevention policies in China.

Additionally, significantly different results were obtained previously by the same as well as by different estimation methods. Therefore, choosing a method providing reliable results is quite challenging. In general, the pollution production coefficients in in different studies are comparable. However, the external conditions such as the pollution treatment method and pollution treatment rate associated with the pollutants transported from the source to the discharge areas differ, thereby producing varying pollution discharge and coefficients of pollution flowing into river. Consequently, obtaining accepted export coefficients of pollutant discharge and pollution into river remains difficult.

Parameters reflecting the policies of agricultural non-point source pollution prevention such as the fertilizer utilization rate, comprehensive utilization rate of livestock and poultry breeding, and treatment rate of rural domestic wastewater were easier to obtain for a specific region. These parameters usually reflect the potential quantity of agricultural non-point source pollution into the environment and the effects of implementing pollution prevention policies. Therefore, using easy accessed coefficients of pollution production and parameters reflecting the prevention policies for assessing the potential agricultural non-point source pollution may be another option.

The main objectives of this study are to estimate the potential quantity of agricultural non-point source pollution into the environment in the Baiyangdian Basin and to quantify the effects of prevention policies on such pollution. The spatial distribution of this agricultural non-point source pollution had been given taking county as the basic unit. Then, the environmental protection policies including increasing the fertilizer use efficiency, improving the straw utilization rate, introducing comprehensive utilization rates for livestock and poultry breeding and others during 2016–2020 are evaluated. Additionally, the maximum of the non-point source pollution reduction potential is also discussed. This study can provide a reference for the tendency of non-point source pollution protection policies in the future.

## 2. Materials and methods

### 2.1 Study area

**Fig 1:**
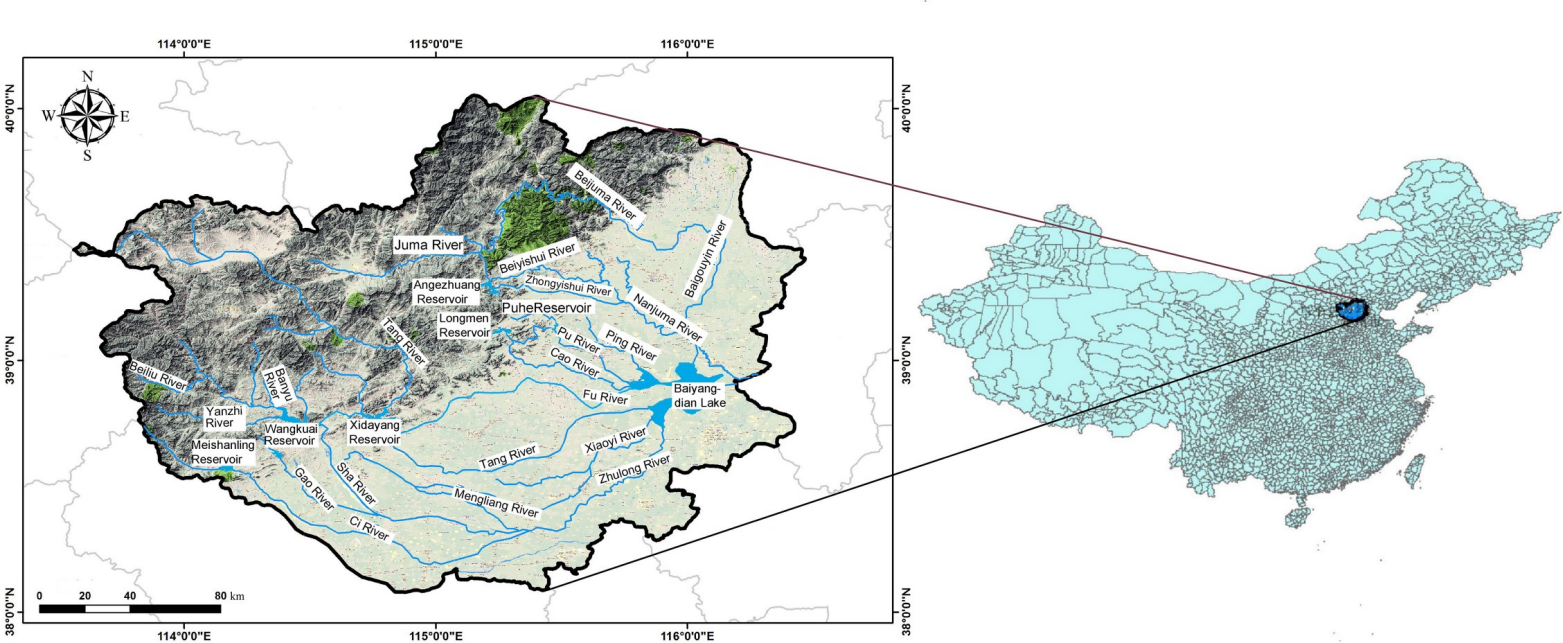
Map of the Baiyangdian Basin and its geographical location in China.

The Baiyangdian Basin is in the middle of the north China plain (113°39'-116°20', 38°39'-40°09'), and belongs to the Daqinghe River system of the Haihe Basin. The watershed covers approximately 3.2×10^6^ ha, with Baiyangdian Lake as the outlet [5]. Baiyangdian Lake is the largest plain lake wetland in north China, which also plays an important role in the construction of the Xiongan New Area. Environment of Baiyangdian Basin has a direct influence on the construction of Xiongan New Area. The topography of the basin is complex including middle mountains, low mountains, hills, plains and lakes from west to east. The basin area is characterized by a semiarid warm temperate monsoon climate, with annual average rainfall of 564 mm, marked by uneven distribution, and showing maximum values from July to August [23]. Agricultural economy is developed in Baiyangdian Basin which is a major grain-producing area in China. The basin crosses 47 prefectures or counties in Hebei province, Shanxi province and Beijing city, including nine rivers of the ZhuLong, Xiaoyi, Tang, Qingshui, Bao, Fu, Cao, Ping rivers as well as Baigou canal [24] displayed in Fig 1. Since the 1970 s, the water quality of rivers in the basin and the Baiyangdian Lake has continued to gradually deteriorate [25]. In fact, in 2014, the Baiyangdian Lake water was described as severely polluted [26]. Although, after intensive management, the COD and NH_3_-N concentrations as well as degree of eutrophication were improved, pollution remains a serious issue in rivers in the basin and the Baiyangdian Lake.

### 2.2 Calculation method

#### 2.2.1 Process of non-point pollution into river

**Fig 2:**
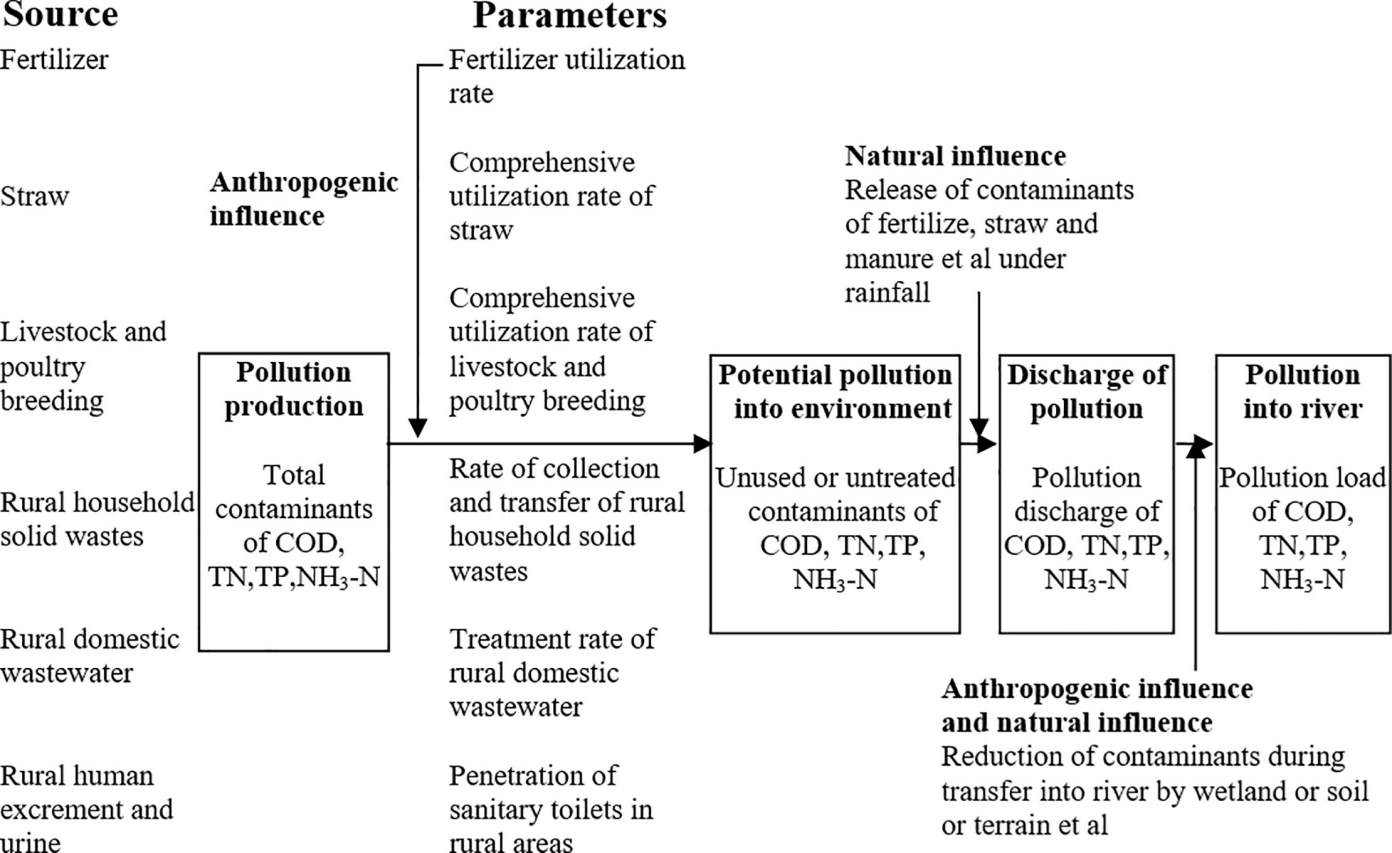
Flowchart summarizing the steps in a typical pollution calculation process.

Pollution from being produced to entering into environment is influenced by natural and human factors [27], and a typical process is displayed in Fig 2. In fact, human control is an effective way to reduce agricultural non-point source pollution [28]. Generally, pollutants production is easily estimated by acknowledged basic calculated parameters such as the pollution production coefficients of rural human excrement and urine, livestock and poultry breeding, chemical fertilizer consumption and rural population and livestock and poultry breeding numbers. Therefore, the potential pollution into an environment can be estimated based on the pollutant production and human influence. As the anthropogenic influence is enormous, most of pollution prevention policies target the activities associated with this stage. Prevention measures include: (1) increasing the fertilizer utilization rate by soil testing and formulated fertilization and integrated irrigation with water and fertilizer method [29]; (2) increasing the comprehensive utilization straw rate by promoting its use as fertilizer, feed, energy, basic material and raw material [30]; (3) improving the comprehensive utilization rate of livestock and poultry breeding by reinforcing harmless treatment and resource utilization of livestock and poultry waste as fertilizer, fodder, and energy etc. through physical and chemical treatment technology [31]; (4) augmenting the capacity of collection and transfer of rural household solid wastes; (5) enhancing treatment rate of rural domestic wastewater by constructing collection networks and increasing rural domestic wastewater disposal facilities; and (6) increasing the penetration of sanitary toilets in rural areas to reduce the pollution caused by rural human excrement and urine directly into environment [32]. The calculation of pollution from expose into environment to flow into river still involves two processes, as depicted in Fig 1.

#### 2.2.2 Calculation method and parameters

The estimation of the potential pollution into environment is based on the export coefficient method, with the county as the basic unit, and chemical fertilizer, straw, livestock and poultry breeding, rural household solid wastes, rural domestic wastewater and rural human excrement and urine were considered as the pollution sources. The equations employed for calculation the potential pollution are showed in Table 1.

**Table 1:** Equations used for estimating the potential pollution of different sources.

Pollution source	Equations	Major pollution index
Chemical fertilizers	*PF*_*i*_ *=* usage of chemical fertilizer *i* × fertilizer utilization rate	TN, TP, NH_3_-N
Straw	*PS*_*i*_ = Σ the *j*^th^ crop yield ×Straw–grain ratio ×production coefficient of pollution index *i*× (1- comprehensive utilization rate of straw)	COD, TN, TP
Livestock and poultry breeding	*PP*_*i*_ = Σ production coefficient of pollution index *i* by the *j*^th^ kinds of poultry × numbers of poultry *j* × (1-comprehensive utilization rate of livestock and poultry breeding)	COD, TN, TP, NH_3_-N
Rural household solid wastes	*PRS*_*i*_ = Σ production coefficient of pollution index *i* × numbers of rural population × 0.8^a^ × (1-rate of collection and transfer of rural household solid wastes)	COD, TN, TP
Rural domestic wastewater	*PRW*_*i*_ = Σ production coefficient of pollution index *i* × numbers of rural population × 0.8^a^ × (1-treatment rate of rural domestic wastewater)	COD, TN, TP, NH_3_-N
Rural human excrement and urine	*PRE*_*i*_ = Σ production coefficient of pollution index *i* × numbers of rural population ×0.8^a^ × (1-penetration of sanitary toilets in rural areas)	COD, TN, TP

*1*. *PF*_*i*_, *PS*_*i*_, *PP*_*i*_, *PRS*_*i*_, *PRW*_*i*_ and *PRE*_*i*_ mean the potential pollution of the pollution index *i* produced by chemical fertilizers, straw, livestock and poultry breeding, rural household solid wastes, rural domestic wastewater, rural human excrement and urine respectively.

2. a is the conversion coefficient from rural permanent population to the rural population [33].

The basic of rural population, livestock and poultry breeding, chemical fertilizer usage and crop yield data were obtained from statistics yearbooks. Parameters including straw–grain ratio and the production coefficients of pollution indexes for different pollution sources were easily obtained from previous studies. The production coefficient of pollutant represents the average pollution production per unit product under regular technical, economic and management conditions. Information for other parameters including the fertilizer utilization rate, comprehensive utilization straw rate, comprehensive utilization rate of livestock and poultry breeding, rate of collection, transfer of rural household solid wastes, treatment rate of rural domestic wastewater and penetration of sanitary toilets in rural areas could be easily obtained from government websites, reports, and news.

In this study, the statistics yearbooks of the cities of Baoding, Langfang, Shijiazhuang, Zhangjiakou, Changzhou, Datong, Qizhou had been used [34–40]. The amount of TN and TP in fertilizer equated to the sum of nitrogenous fertilizers and 0.33 times of compound fertilizer usage, the sum of 0.44 times of phosphate fertilizer and 0.15 times of compound fertilizer usage in the statistical yearbooks, respectively. The usage of NH_3_-N was 8.3% of that of the TN [41]. The straw-grain ratio and pollutant production coefficients of different crops straws are showed in Table 2 [42], whereas for the rural domestic wastewater, the pollutant production coefficients of COD, NH_3_-N, TN and TP were 5.237, 1.278, 1.599 and 0.142 kg yr^-1^ per person [20]. Additionally, the pollutant production coefficients of COD, TN and TP for rural human excrement and urine were 0.054, 0.084 and 0.014 Mg yr^-1^ per person, respectively [43]. The pollutant production coefficients of livestock and poultry breeding are showed in Table 3 [41].

**Table 2:** Straw–grain ratios and pollutant production coefficients of straws from different crops.

	Rice	Wheat	Corn	Millet	Beans	Mung bean	Red bean	Tuber crops	Oil crops	Cotton	Vegetable	Melon and fruit
Straw–grain ratio	0.90	0.97	1.03	1.60	1.60	2.00	2.00	0.61	3.00	3.40	0.10	0.10
COD/ kg Mg^-1^	5.63	6.39	11.23	5.63	17.61	17.61	17.61	2.26	20.57	11.23	5.10	5.10
TN/ kg Mg^-1^	5.82	5.15	10.69	5.82	22.23	22.23	22.23	1.83	45.43	10.69	0.92	0.92
TP/ kg Mg^-1^	0.42	0.90	2.39	0.42	2.24	2.24	2.24	0.67	3.06	2.39	0.45	0.45

**Table 3:** Discharge coefficients for different livestock and poultry (kg head^-1^).

Livestock and poultry species	Sheep	Pig	Cattle	Poultry
COD	8.89	36.00	712	0.99
NH_3_-N	0.14	1.80	2.52	0.02
TN	8.82	31.73	104.10	1.85
TP	1.88	4.22	10.17	0.48

### 2.3 Policy influence

To control and prevent agricultural non-point source pollution, several effective measures have been done by government, such as integrated irrigation with water and fertilizer promotion, sustainable farming promotion, formulating the development plan of regional livestock and poultry breeding according to environmental protection, setting standards for sewage discharge standard, and creating agricultural circular ecological economy. Under the different policies of environmental protection, data for relevant coefficients obtained from government websites or reports are presented in Table 4. In Baoding city, the average of fertilizer used per unit cultivated cropland is approximately 590 kg ha^-1^ which is over 4 times the global average level and even in Jingxiu and Lianchi district the values were more than 1000 kg ha^-1^. The potential for reducing chemical fertilizer usage is high, and hence, we had taken the usage of chemical fertilizer reduced 20% as the assumptive situation.

**Table 4:** Relevant coefficients associated with different policies (%).

	FUR	CURS	CURL	RCTRS	TRDW	PST
2016	35.2	95.6	65	80	22	52.3
2018	37.8	96.8	68	90	26	64.0
2019	39.2	96.8	75	95	27	74.0
2020	40.0	96.8	70	100	38	85.0
Assumption 1^b^	50.0	98.0	80	100	70	90.0
Assumption 2	Usage of chemical fertilizer reduced 20%, other parameters were same with Assumption 1					

a FUR, CURS, CURL, RCTRS, TRDW and PST mean fertilizer utilization rate, comprehensive utilization rate of straw, comprehensive utilization rate of livestock and poultry breeding, rate of collection and transfer of rural household solid wastes, treatment rate of rural domestic wastewater and penetration of sanitary toilets in rural areas respectively.

b Assumption 1 is based on the future of pollution control target of the government.

## 3. Results

### 3.1 Total potential pollution

**Fig 3:**
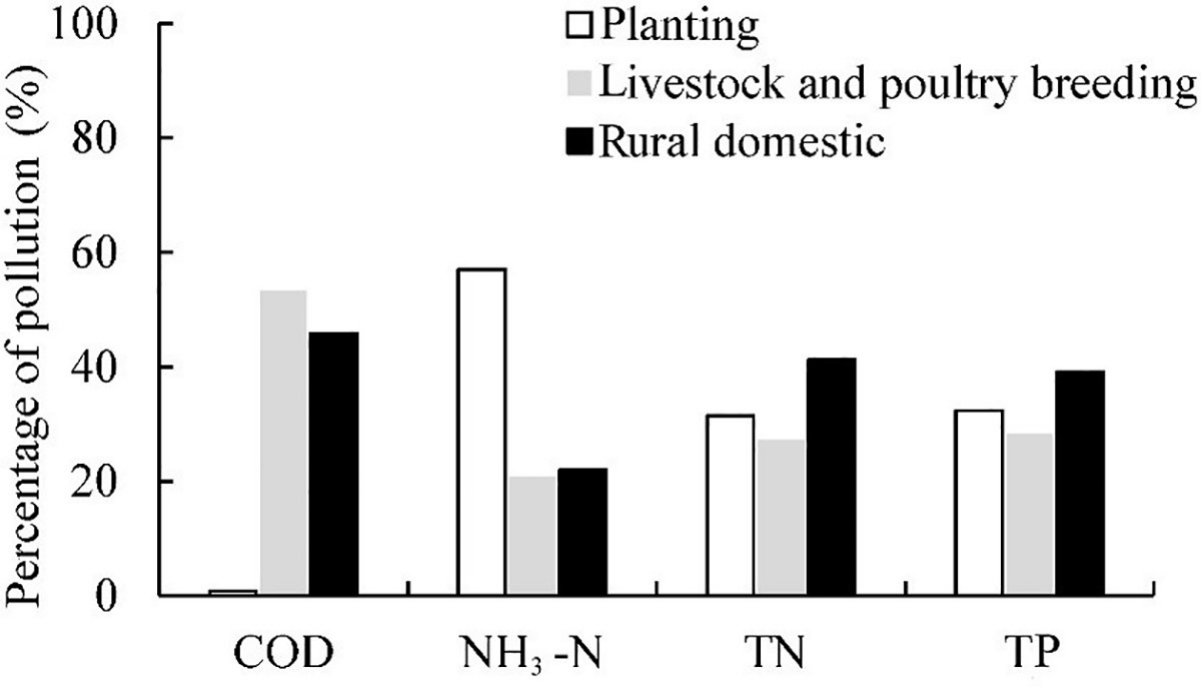
The contribution percentages of different potential pollution sources.

According to the estimation method employed and relevant parameters for 2016, the potential quantity of agricultural non-point source pollution of COD, NH_3_-N, TN and TP are 60.89×10^4^, 3.93×10^4^, 87.05×10^4^, and 15.10×10^4^ Mg, respectively, for the Baiyangdian Basin. Evidently, the TN represents the highest threat of agricultural non-point source pollution, followed by the COD. The relative pollution contributions of different pollution sources are displayed in Fig 3. We could see that all of planting, the livestock and poultry breeding, rural domestic had big threats of pollution. The livestock and poultry breeding produces approximately 53%, 21%, 27%, and 28% of the total potential pollution (total potential pollutants produced by all sources) of COD, NH_3_-N, TN, and TP, respectively. The rural domestic source produced the highest potential pollution of TN and TP with 41% and 39% of the total pollution, respectively, while planting accounts for the highest potential pollution of NH_3_-N with 57%. Pollution from livestock and poultry breeding and rural domestic source are comparable for COD pollution, whereas TN and TP pollution by these sources are low. For comparison with previous studies, the pollution intensity, reflecting the pollution per unit area has been considered. In 2016, the potential pollution intensities of COD, NH3-N, TN and TP are 190, 12, 272, and 47 kg ha^-1^, respectively, which are within results of literatures [19, 20].

### 3.2 Spatial distribution of potential pollution

**Fig 4:**
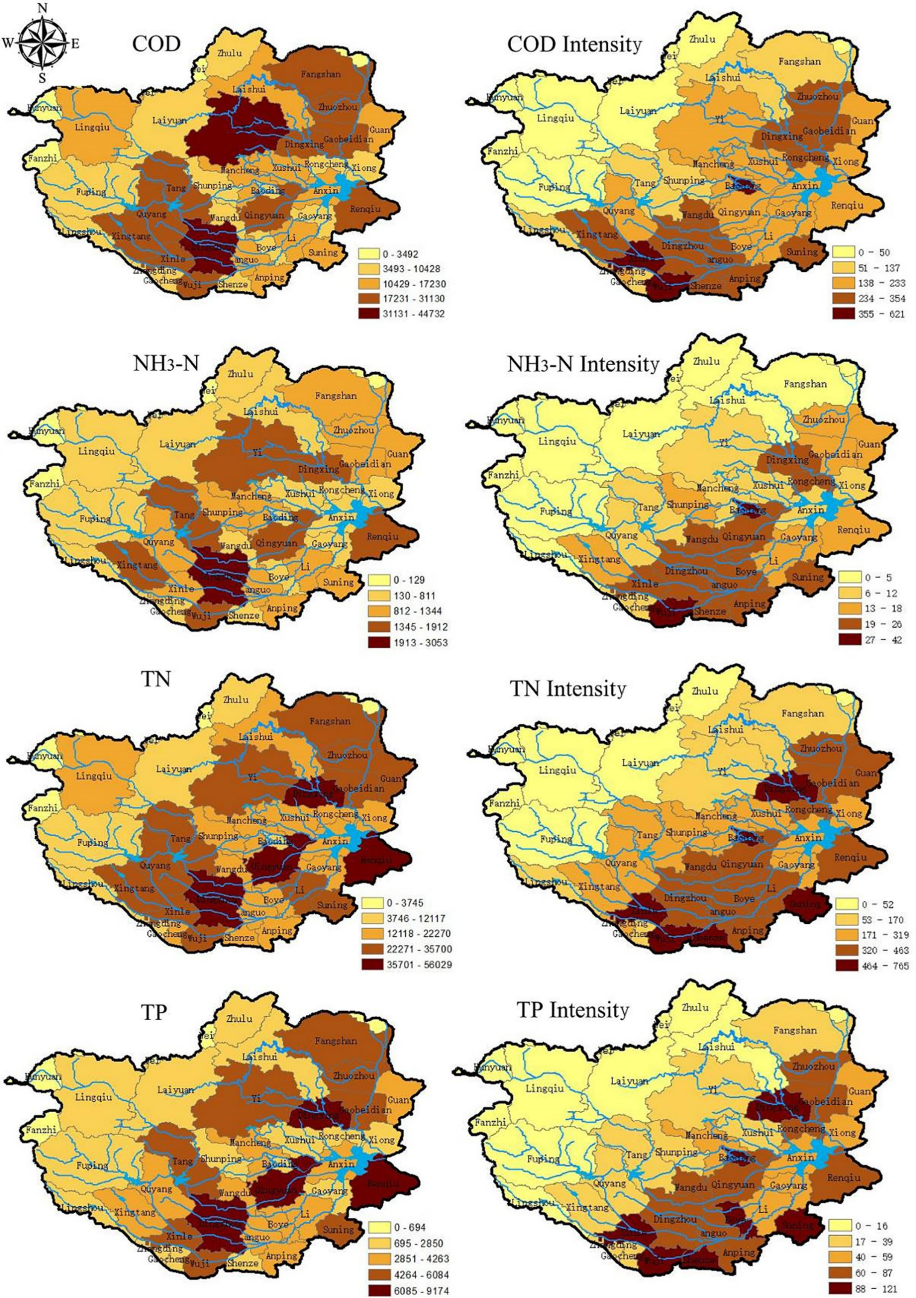
Spatial distribution of total potential pollution (Mg) and intensities in the Baiyangdian Basin (kg/ha).

The spatial distribution of the calculated total potential pollution and pollution intensities in Baiyangdian Basin are shown in Fig 4. Generally, the counties with high agricultural non-point source pollution produce presented a northeast to southwest direction which was same as the alignment of Taihang mountains west of the basin. Consequently, the counties with high pollution intensities are common in the south and east portion of the basin.

Total potential pollution of COD in Dingzhou city, Yi, and Wuji county accounts for the top three, while values in the west of the basin are low, and the top areas for pollution intensities are the Zhengding, Wuji counties and central Baoding city (Table 5). It is evident that high total potential pollution always affects the results for an area and the intensities. In the Zhengding, Wuji counties and central of Baoding city, the livestock and poultry breeding per unit area are much higher, thereby accounting for the higher pollution intensities.

**Table 5:** Classification of counties with higher pollution intensity of different pollutants (Kg/ha).

Order	COD		NH3		TN		TP	
	County /District	Intensity	County /District	Intensity	County /District	Intensity)	County /District	Intensity
1st	Wuji	666	Jingxiu	53	Jingxiu	1070	Jingxiu	173
2nd	Zhengding	625	Zhengding	38	Zhengding	795	Zhengding	142
3rd	Jingxiu	609	Lianchi	34	Wuji	734	Gaocheng	141
4th	Gaocheng	481	Wuji	33	Gaocheng	696	Wuji	130
5th	Xinle	435	Gaocheng	30	Xinle	597	Dingxing	117

For the NH_3_-N pollution, the top three total potential pollution were produced in Dingzhou city, Qingyuan, Xushui districts, whereas higher pollution intensities are observed in central of Baoding city and the Zhengding county. These observations are likely due to the very high usages of fertilizer per unit area of cultivated croplands in the Jingxiu and Lianchi districts, which also had shown the highest livestock and poultry breeding intensities in 2016. Compared with the potential pollution intensity of COD, the counties with higher NH_3_-N intensity are close to the Baiyangdian Lake, highlighting a grave threat to the water quality of the lake.

The total TN pollution in the cities of Dingzhou, Dingxing as well as the Wuji county emerge as the top three highest, whereas the TN pollution intensities are highest in the Jingxiu district, Wuji and Zhengding counties. The TN pollution is considered a comprehensive effect of planting, livestock and poultry breeding, and rural domestic activities. Besides, higher TN pollution production also occurs near the Baiyangdian Lake. The pollution distribution of TP was similar with that of TN.

### 3.3 Policy influence on potential pollution

**Fig 5:**
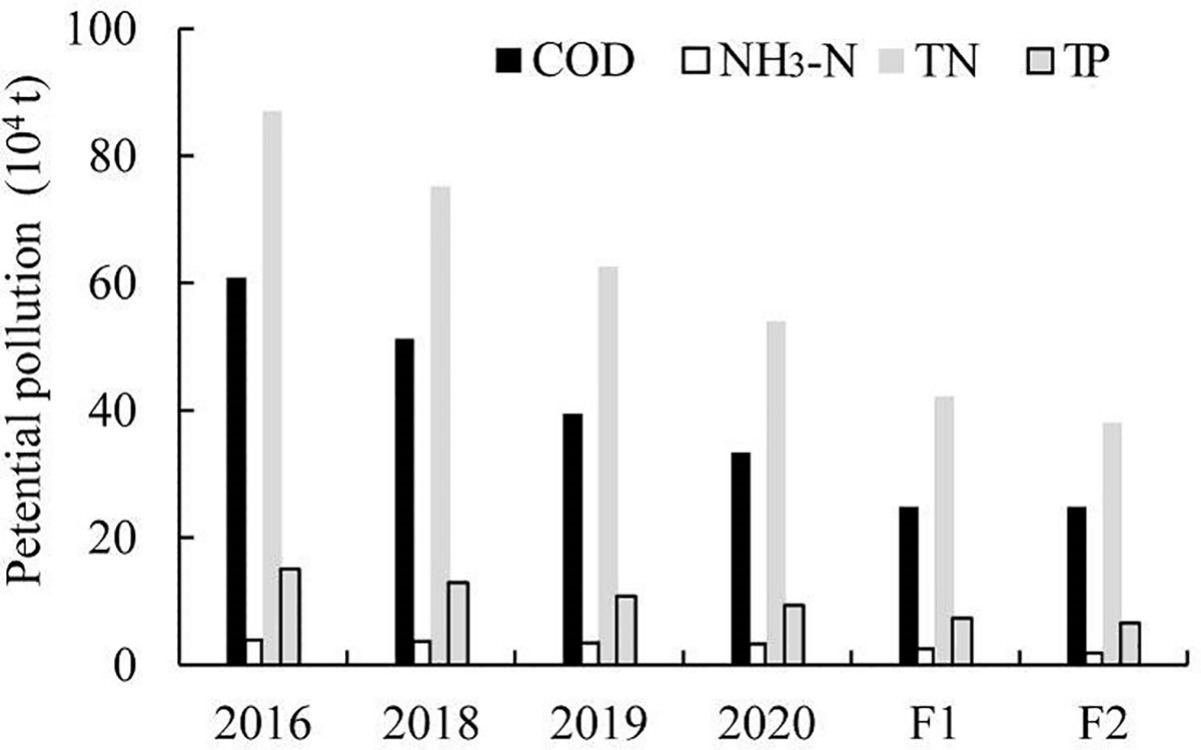
Potential pollution under different policies.

The potential pollution under different policies are exhibited in Fig 5, with F1 and F2 representing the cases under *Assumptions* 1 and 2. Clearly, under the control and prevention policies, the total potential pollution decreases gradually. Compared with 2016, total potential pollution of COD, NH_3_-N, TN and TP in 2020 will decrease by 45.1%, 14.7%, 37.9%, and 37.4%, respectively. Considering consistent measures, when the coefficients increase to the conditions of *Assumption* 1, the total potential pollution of COD, NH_3_-N, TN, and TP decrease 25.7%, 24.4%, 21.7% and 21.6%, respectively, relative to the conditions in 2020. If the usage of chemical fertilizer reduces by 20% on basis of F1 case, then the total potential pollution of COD, NH_3_-N, TN, and TP decrease by 0%, 24.6%, 9.9%, and 9.7% respectively, compared with the conditions of case F1, and are 59.0%, 51.4%, 56.2%, and 55.7% lower, respectively, than the values under the 2016 conditions. That is to say the persistent police application have great effect on reducing the pollution continuously and chemical fertilizer reduction is an effective measure with great potential for decreasing the pollution.

**Fig 6:**
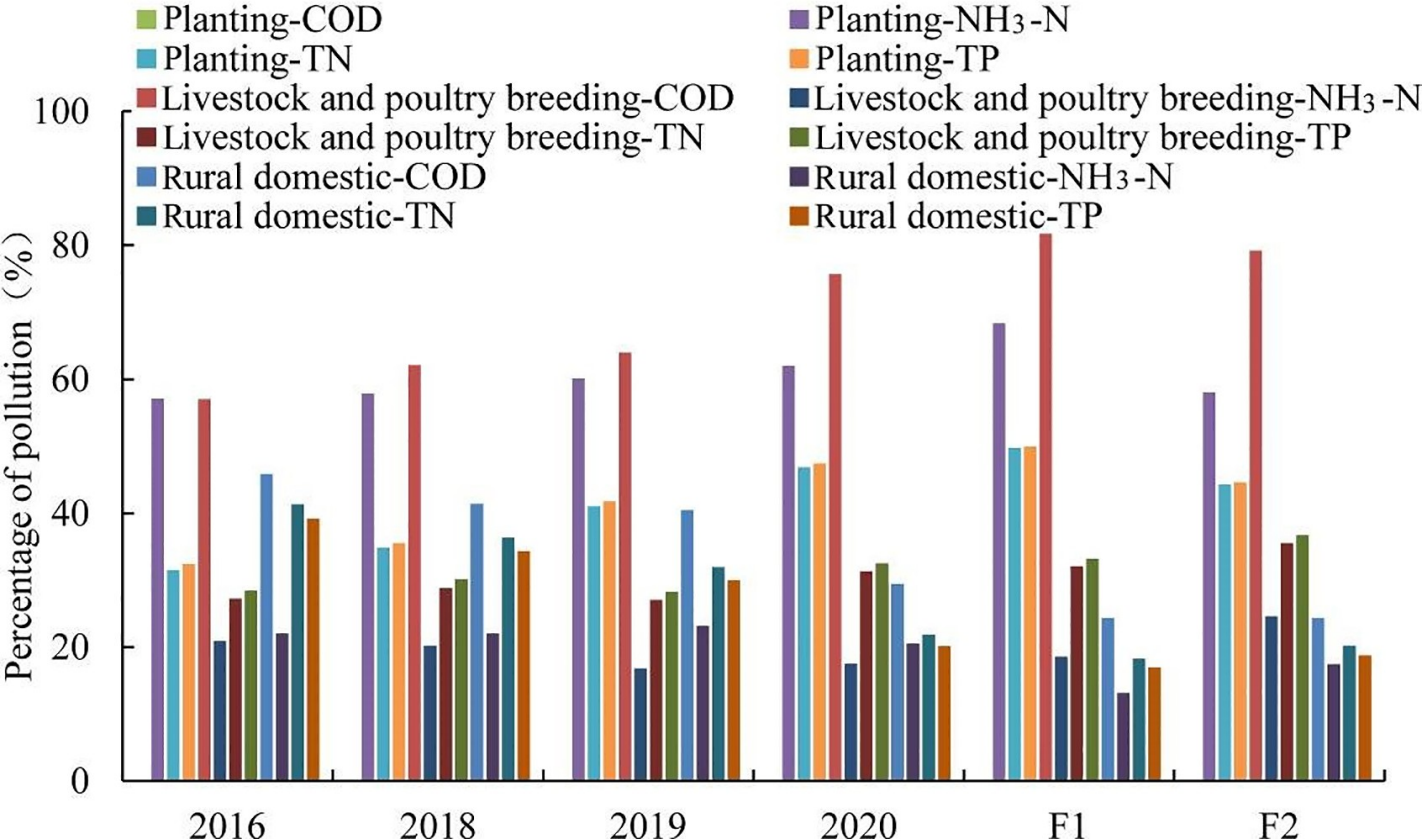
Percentages of potential pollution under different policies.

Additionally, percentages of potential pollution under different policies are displayed in Fig 6. Compared with 2016, with the application of the prevention policies, it could be seen that the contributions of potential pollution by rural domestic obviously decreased, whereas those by planting and livestock and poultry breeding increase in 2020. That is to say future protection measures should focus on livestock and poultry breeding as well as planting in the future.

### 3.4 Contribution to the pollution into river

**Fig 7:**
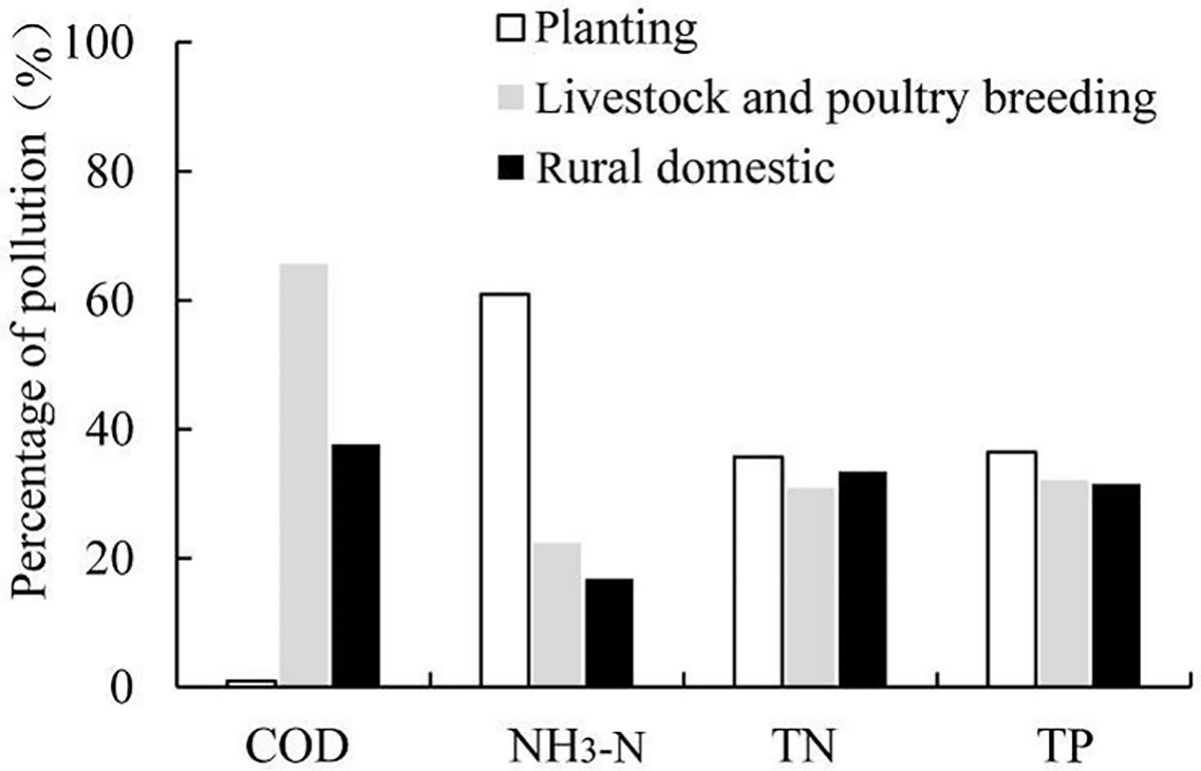
Relative contribution of the pollution into river from different pollution source in Baiyangdian Basin.

The potential pollution represents the total pollution into the environment including water, soil, and the atmosphere. Therefor, pollutants amount into water was only a fraction of potential pollution. Water quality is an immediate response to the water pollution. According to Fig 1, we can find that some potential pollution flow into river through contaminants release and transport. This process should provide coefficients of pollution into the river, although these are hardly obtained precisely. Based on existing coefficients of pollution into rivers, we estimated the pollution into river. The coefficients of pollution into rivers for planting, livestock and poultry breeding, and rural domestic source are 0.07, 0.07, and 0.05, respectively [44]. Based on calculations, the pollution of COD, NH_3_-N, TN and TP into rivers are 37041, 2577, 53749, and 9384 Mg, respectively. According to the data in Fig 7, the relative water pollution contribution into water from different sources agrees with that of potential pollution. The effect of livestock and poultry breeding obviously increases.

These results highlight a significant potential for agricultural non-point source pollution reduction. Protection policies should focus on continuously improving the relevant coefficients for pollution management. In fact, the pollution protection policies for livestock and poultry breeding require reinforcement.

## 4. Discussion

To unify the quantified pollution indexes, an equivalent pollution load method that provides a more obvious evaluation of the threat to the environment pollution was employed[45, 46]. The equivalent pollution load equals to the sum of different pollution loads divided by the limits of the surface water environment quality standard respectively. Similarly, we introduced the concept of equivalent potential pollution to comprehensively reflect the influence of the potential pollution on environment. The water quality target of Baiyangdian Lake is to satisfy the class III of the surface water environment quality standard, with the limit concentrations of COD, NH_3_-N, TN and TP of 5, 1, 1, and 0.2 mg L^-1^ respectively. In 2016, 2018, 2019, and 2020, the calculated equivalent potential pollutions are 17.8, 15.4, 12.8, and 11.1 billion m^3^, respectively. Therefore, we could see that the comprehensive influence of the potential pollution on environment in 2020 is approximately 38% lower than that in 2016. In order to understand the most influential coefficient on equivalent potential pollutions, the SPSS software was been used. Based on principal component analysis, the fertilizer utilization rate emerges as the most influential. In China, the fertilizer utilization rate has a great space for improving. More polices can focus on improving fertilizer utilization rate in future.

Additionally, compared with the government’s actions, the market behaviors associated with non-point source pollution protection are still at a rudimentary stage in China. To further reduce non-point source pollution, attention on the market behaviors is also required. The emission rights trading system and third-party governance relative to pollutants are rarely pursued despite their enormous potential. The emission right trading system involves the trading between point source pollution and non-point source pollution as well as trading within non-point pollution sources [47, 48]. Because the cost of point source pollution control exceeds that of non-point source pollution, trading the former can induce point source pollution production to control agricultural non-point source pollution and augment more permit of emissions [49]. The trading within point source pollution can also trigger the adoption of new techniques and methods for the associated production activities [50]. As we all know that straws, livestock and poultry breeding wastes, and rural domestic wastes are known resources that can benefit the economy. Additionally, third-party governance and social capital can be introduced to effectively control agricultural non-point source pollution [49]. Moreover, ecological and circular agriculture is the most effective way to solve the problem of agricultural non-point source pollution and this requires the introduction of social capital.

## 5. Conclusions

The potential quantity of agricultural non-point source pollution into environment instead of pollution load in the Baiyangdian Basin was estimated in this study by a simple method, with considering the effects of different policies on agricultural non-point source pollution control and prevention. The spatial distribution of the agricultural non-point source pollution was studied taking county as basic unit. The main conclusions from this study are as follows. Firstly, in the Baiyangdian Basin, TN pollution posed the highest potential threat of agricultural non-point source pollution, followed by COD. Livestock and poultry breeding produced the largest potential pollution of COD, while the rural domestic source created the most potential pollution of TN and TP, and planting source produced the highest potential pollution of NH_3_-N. Secondly, spatially, the counties with the highest non-point source pollution presented a northeast to southwest direction, consistent with the Taihang mountain alignment. The total potential pollution of the Dingzhou and the pollution intensities of the Zhengding county were very large for all pollution indexes of COD, NH_3_-N, TN, and TP. Thirdly, under the policies of agricultural non-point source pollution control and prevention, the total potential pollution gradually decreased from 2016 to 2020. Compared with 2016, the contributions of potential pollution by rural domestic sources obviously decreased, whereas those by planting and livestock and poultry breeding increased in 2020. Besides, the protection policies should consist and should be strengthen for livestock and poultry breeding as well as planting. Finally, future polices can focus on improving the fertilizer utilization rate and considering comprehensive influence of potential pollution on environment. Third-party governance and social capital can be introduced to effectively control the agricultural non-point source pollution.
